# Angiotensin II-induced cardiac hypertrophy and fibrosis are promoted in mice lacking *Fgf16*

**DOI:** 10.1111/gtc.12055

**Published:** 2013-04-18

**Authors:** Emi Matsumoto, Sayaka Sasaki, Hideyuki Kinoshita, Takuya Kito, Hiroya Ohta, Morichika Konishi, Koichiro Kuwahara, Kazuwa Nakao, Nobuyuki Itoh

**Affiliations:** 1 Department of Genetic Biochemistry, Kyoto University Graduate School of Pharmaceutical SciencesSakyo, Kyoto, 606-8501, Japan; 2 Department of Medicine and Clinical Science, Kyoto University Graduate School of MedicineSakyo, Kyoto, 606-8507, Japan

## Abstract

Fibroblast growth factors (Fgfs) are pleiotropic proteins involved in development, repair and metabolism. *Fgf16* is predominantly expressed in the heart. However, as the heart function is essentially normal in *Fgf16* knockout mice, its role has remained unclear. To elucidate the pathophysiological role of Fgf16 in the heart, we examined angiotensin II-induced cardiac hypertrophy and fibrosis in *Fgf16* knockout mice. Angiotensin II-induced cardiac hypertrophy and fibrosis were significantly promoted by enhancing *Tgf-β*_*1*_ expression in *Fgf16* knockout mice. Unexpectedly, the response to cardiac remodeling was apparently opposite to that in *Fgf2* knockout mice. These results indicate that Fgf16 probably prevents cardiac remodeling, although Fgf2 promotes it. Cardiac *Fgf16* expression was induced after the induction of *Fgf2* expression by angiotensin II. In cultured cardiomyocytes, *Fgf16* expression was promoted by Fgf2. In addition, Fgf16 antagonized Fgf2-induced *Tgf-β*_*1*_ expression in cultured cardiomyocytes and noncardiomyocytes. These results suggest a possible mechanism whereby Fgf16 prevents angiotensin II-induced cardiac hypertrophy and fibrosis by antagonizing Fgf2. The present findings should provide new insights into the roles of Fgf signaling in cardiac remodeling.

## Introduction

Fibroblast growth factors (Fgfs), proteins of ∼150–300 amino acids, play diverse roles in development, repair and metabolism. The human/mouse Fgf family comprises twenty-two members (Itoh & Ornitz 2008, 2011). Most Fgfs mediate biological responses by binding to and activating Fgf receptors (Fgfrs) in a paracrine manner (Beenken & Mohammadi 2009; Itoh & Ornitz 2011). Among paracrine Fgfs, *Fgf16* is predominantly expressed in the heart. *Fgf16* expression is weakly detected in the embryonic heart and much more abundant at adult stages than embryonic stages. These findings indicate potential roles in the heart (Hotta *et al*. 2008; Lu *et al*. 2008a; Fon Tacer *et al*. 2010). Three lines of *Fgf16* knockout mice have been reported. Two of the lines are viable and fertile. Although the proliferation of embryonic cardiomyocytes temporarily decreases in our *Fgf16* knockout mice on a C57BL/6 background around embryonic day (E) 14.5, the heart function is essentially normal in *Fgf16* knockout mice (Hotta *et al*. 2008). The cardiac phenotype of the other *Fgf16* knockout mice on a 129/B6 background has not been reported (Hatch *et al*. 2009). In contrast, *Fgf16* knockout mice on a Black Swiss background died at around E11.5, indicating that Fgf16 is required for embryonic heart development in midgestation (Lu *et al*. 2008a). The phenotypes are potentially affected by genetic backgrounds (Lu *et al*. 2010).

As the heart function is essentially normal in *Fgf16* knockout mice, the role of Fgf16 in the heart remains unclear (Hotta *et al*. 2008). In hypertension, the heart responds to increased afterload by initiating adaptive remodeling processes including cardiac hypertrophy and fibrosis. Although *Fgf2* is broadly expressed in mice, hypertension-induced cardiac hypertrophy and fibrosis are less developed in *Fgf2* knockout mice, indicating that Fgf2 promotes them (Virag *et al*. 2007; House *et al*. 2010). From these findings, we expected that Fgf16 also might play pathophysiological roles in the heart. The renin–angiotensin system is a key mediator of cardiac adaptations to hemodynamic overload. Angiotensin II induces hypertension and cardiac hypertrophy and fibrosis (Rosenkranz 2004). To elucidate the pathophysiological role of Fgf16 in the heart, we examined angiotensin II-induced cardiac hypertrophy and fibrosis in *Fgf16* knockout mice. Unexpectedly, possible adaptive remodeling processes were significantly promoted, indicating that the role of Fgf16 is apparently distinct from that of Fgf2. Here, we report a possible mechanism whereby Fgf16 prevents angiotensin II-induced cardiac hypertrophy and fibrosis.

## Results

### Compensatory cardiac response to angiotensin II is promoted in *Fgf16* knockout mice

**Figure 1:**
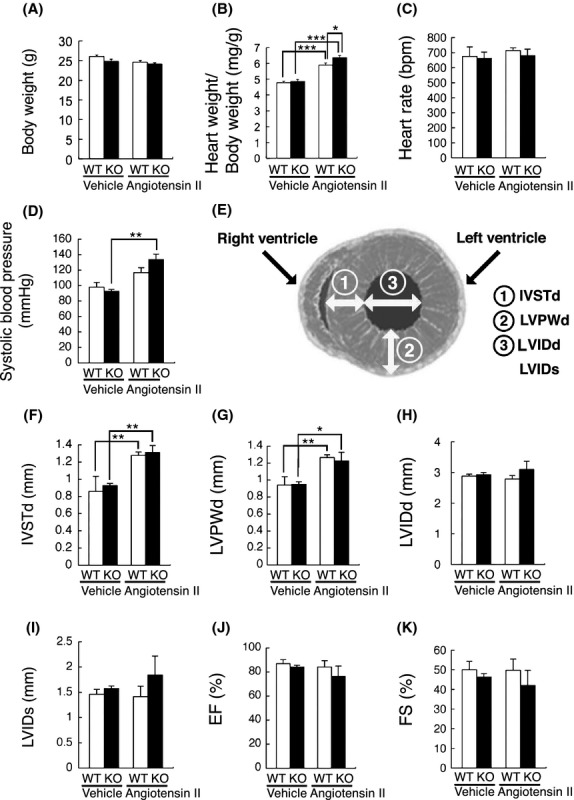
Body and heart weights, systolic blood pressure and echocardiographic parameters. Body and heart weights (A, B), heart rate (C), systolic blood pressure (D), a schematic representation of cross-sectional cardiac anatomy (E), interventricular septal thickness diastolic (IVSTd) (F), left ventricular end posterior wall dimension diastolic (LVPWd) (G), left ventricular internal dimension diastolic (LVIDd) (H), left ventricle internal dimension systolic (LVIDs) (I), ejection fraction (EF) (J) and fractional shortening (FS) (K) were examined in both wild-type and *Fgf16* knockout mice infused with either vehicle or angiotensin II. Results are expressed as the mean ± SEM for mice infused with vehicle (wild type, *n* = 3–11; *Fgf16* knockout, *n* = 4–7) or angiotensin II (wild type, *n* = 7–15; *Fgf16* knockout, *n* = 5–14). Asterisks indicate statistical significance (*P < 0.05; **P < 0.01; ***P < 0.001).

We examined body and heart weights of wild-type and *Fgf16* knockout mice (Fig. 1A,B). Although body weight was essentially unchanged in the mice infused with angiotensin II for 14 days, heart weight was significantly increased. The *Fgf16* knockout mice had slightly but significantly heavier hearts than the wild-type mice. We also examined systolic blood pressure and echocardiographic parameters. Heart rate was essentially unchanged in both groups. However, systolic blood pressure tended to be increased in the wild-type mice and was significantly increased in the knockout mice (Fig. 1C,D). Interventricular septal thickness diastolic (IVSTd) and left ventricular end posterior wall dimension diastolic (LVPWd) were significantly increased in both groups. However, IVSTd and LVPWd in the knockout mice were similar to those in the wild-type mice (Fig. 1E–G). In contrast, left ventricular internal dimension diastolic (LVIDd) and left ventricle internal dimension systolic (LVIDs) were essentially unchanged in the wild-type mice, whereas they tended to be slightly increased in the knockout mice (Fig. 1E,H,I). Ejection fraction (EF) represents the volumetric fraction of blood pumped out of the heart with each heartbeat. Fractional shortening (FS) is used as an estimate of myocardial contractility. EF and FS were also essentially unchanged in the wild-type mice, but they tended to be slightly decreased in the knockout mice (Fig. 1J,K). These results suggest a possible compensatory cardiac response to angiotensin II is promoted in *Fgf16* knockout mice.

### Angiotensin II-induced cardiac hypertrophy and fibrosis are promoted in *Fgf16* knockout mice

**Figure 2:**
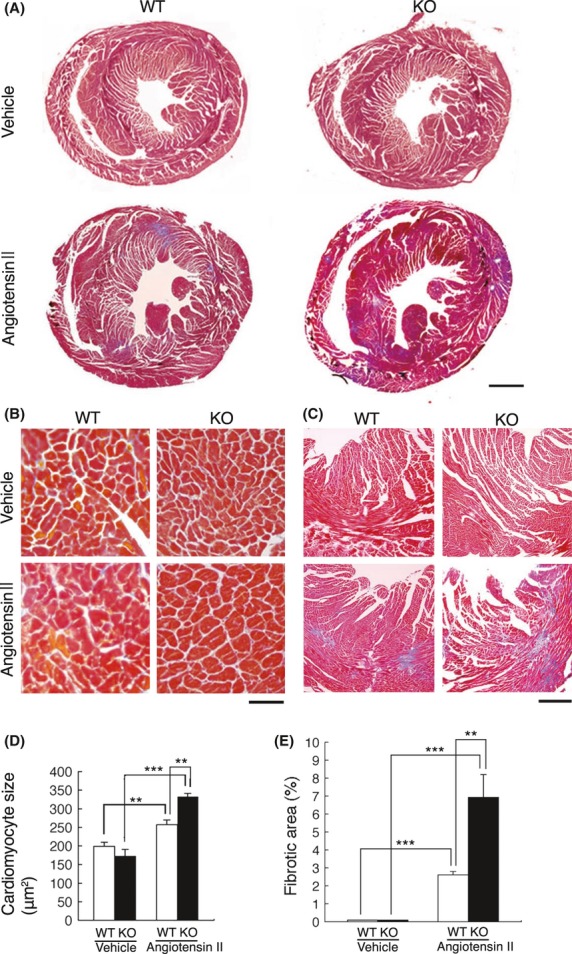
Cardiac hypertrophy and fibrosis. Sections of the heart were stained with Masson's trichrome (A). The size of cardiomyocytes in the section of left ventricular end posterior wall (LVPW) was determined from the cells' cross-sectional area (B, D). Blue-stained interstitial fibrotic areas in the sections were quantitatively determined (C, E). Results are expressed as the mean ± SEM for mice infused with vehicle (wild type, *n* = 5–7; *Fgf16* knockout, *n* = 4–6) or angiotensin II (wild type, *n* = 6–14; *Fgf16* knockout, *n* = 4–12). Asterisks indicate statistical significance (**P < 0.01; ***P < 0.001). Scale bars = 1 μm (A), 50 μm (B) and 300 μm (C).

Cardiac hypertrophy represents an adaptive process of the heart in response to work overload (Berk *et al*. 2007). Sections of heart stained with Masson's trichrome were examined by light microscopy (Fig. 2A). The size of cardiomyocytes was examined by determining the cells' cross-sectional area in LVPW (Fig. 2B,D). The size was significantly increased in both wild-type and *Fgf16* knockout mice infused with angiotensin II. However, it was significantly larger in the knockout mice. Cardiac remodeling is also associated with increased numbers of fibroblasts in the myocardium (Berk *et al*. 2007). Cardiac fibrosis is characterized by the increased deposition of extracellular matrix components and proliferation of interstitial fibroblasts. Extended fibrosis results in increased myocardial stiffness, causing ventricular dysfunction and ultimately heart failure (Weber & Brilla 1991). Interstitial fibrotic areas were stained with blue dye and quantitatively determined (Fig. 2C,E). The areas were markedly increased in both groups infused with angiotensin II. However, they were significantly larger in the knockout mice.

### Cardiac expression of genes related to cardiac remodeling is promoted in *Fgf16* knockout mice

**Figure 3:**
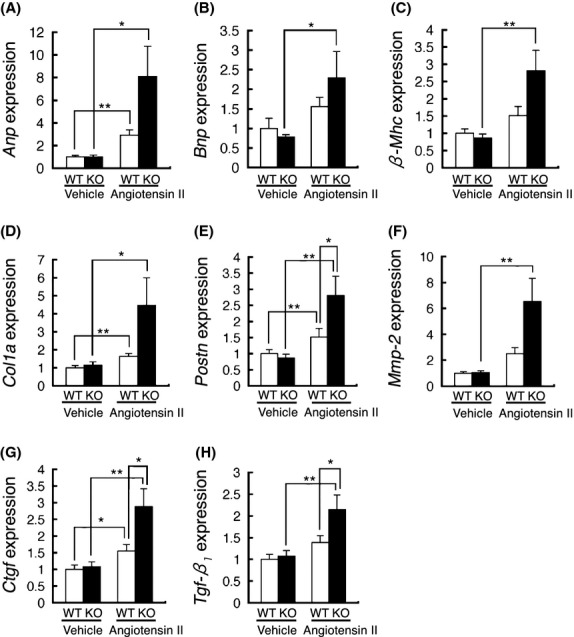
Cardiac expression of genes related to cardiac hypertrophy and/or fibrosis. Cardiac expression of their genes (A–H) was examined by RT-qPCR. Results are expressed as the mean ± SEM for mice infused with vehicle (wild type, *n* = 10; *Fgf16* knockout, *n* = 7) or angiotensin II (wild type, *n* = 16; *Fgf16* knockout, *n* = 15). Asterisks indicate statistical significance (*P < 0.05; **P < 0.01).

Atrial natriuretic peptide (Anp) and brain natriuretic peptide (Bnp) are cardiac endocrine hormones/paracrine factors. *Anp* and *Bnp* expression levels are increased in the heart with cardiac hypertrophy and fibrosis (Nishikimi *et al*. 2006). β-Myosin heavy chain (βMhc) is one of the Mhc isoforms. *βMhc* expression levels are also increased in cardiac hypertrophy (Morkin 2000). We examined *Anp*, *Bnp* and *βMhc* expression in the heart by reverse transcription–quantitative polymerase chain reaction (RT-qPCR) (Fig. 3A–C). *Anp, Bnp* and *βMhc* expression levels were significantly or tended to be increased in both wild-type and *Fgf16* knockout mice infused with angiotensin II. Their levels tended to be higher in the knockout mice. Collagen type 1a (Col1a) is often defined as a component of extracellular matrices (Exposito *et al*. 2010). Connective tissue growth factor (Ctgf) is a matricellular protein that promotes angiogenesis. Periostin (Postn) is a secreted extracellular matrix protein belonging to the fasciclin family (Conway & Molkentin 2008). Matrix metallopeptidase 2 (Mmp2) plays a key role in matrix turnover (Stamenkovic 2003). Their expression is induced in hearts with cardiac fibrosis (Bergman *et al*. 2007; Nishida *et al*. 2008; Leask 2010). Their cardiac expression levels were significantly or tended to be increased in both groups infused with angiotensin II (Fig. 3D–G). Their levels were significantly or tended to be higher in the knockout mice. These results support those of the histochemical analysis.

Transforming growth factor-β_1_ (Tgf-β_1_) is also a key mediator of cardiac adaptations to hemodynamic overload and thus critically involved in the pathogenesis of cardiac hypertrophy and fibrosis. Tgf-β_1_ acts downstream of angiotensin II and promotes angiotensin II-induced cardiac hypertrophy and fibrosis (Rosenkranz 2004). Cardiac *Tgf-β*_*1*_ expression levels were increased in both mice infused with angiotensin II (Fig. 3H). In addition, its levels were significantly higher in the knockout mice.

### Fgf16 antagonizes Fgf2-induced *Tgf-β_1_* expression in cultured cardiomyocytes and noncardiomyocytes

**Figure 4:**
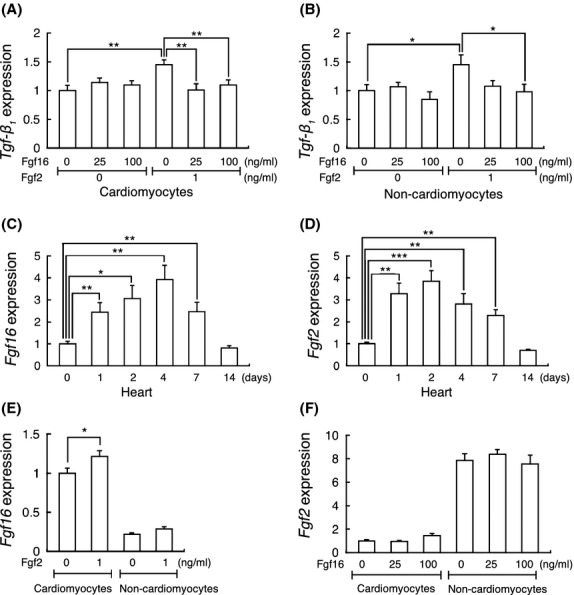
Effects of Fgf16 and Fgf2 in cultured cardiomyocytes and noncardiomyocytes and *Fgf16* and *Fgf2* expression in the heart. The effects of Fgf16 and Fgf2 on *Tgf-β*_*1*_ expression in cultured cardiomyocytes and noncardiomyocytes were examined by RT-qPCR (A, B). Cardiac *Fgf16* and *Fgf2* expression in mice infused with angiotensin II for 1–14 days was examined by RT-qPCR (C, D). The effect of Fgf2 on *Fgf16* expression in cultured cardiomyocytes and noncardiomyocytes were examined by RT-qPCR (E). The effect of Fgf16 on *Fgf12* expression in cultured cardiomyocytes and noncardiomyocytes was examined by RT-qPCR (F). Results are expressed as the mean ± SEM for mice infused with angiotensin II (*n* = 5–15) and the cultured cells (*n* = 11–14). Asterisks indicate statistical significance (*P < 0.05; **P < 0.01; ***P < 0.001).

Lu *et al*. reported that Fgf2 showed significant proliferative activity in cultured neonatal rat cardiomyocytes, but Fgf16 did not. However, Fgf16 antagonized the activity of Fgf2 (Lu *et al*. 2008b). Cultured neonatal rat cardiomyocytes and noncardiomyocytes have been well-established (Nakagawa *et al*. 1995), but mouse cells not. We also examined the effects of Fgf16 and Fgf2 on *Tgf-β*_*1*_ expression in cultured neonatal rat cardiomyocytes and noncardiomyocytes (Fig. 4A,B). Although Fgf2 significantly induced *Tgf-β*_*1*_ expression in both cells, Fgf16 did not. However, Fgf16 repressed Fgf2-induced *Tgf-β*_*1*_ expression, indicating that Fgf16 antagonizes Fgf2-induced *Tgf-β*_*1*_ expression. These results are essentially consistent with the results by Lu *et al*. (Lu *et al*. 2008b).

### Cardiac Fgf16 and Fgf2 expression levels are increased by angiotensin II infusion

We examined cardiac *Fgf16* and *Fgf2* expression in the mice infused with angiotensin II for 1–14 days (Fig. 4C,D). Both *Fgf16* and *Fgf2* expression levels were significantly increased by angiotensin II infusion. *Fgf2* expression levels were maximally increased at 2 days and thereafter gradually decreased. However, *Fgf16* expression levels were maximally increased at 4 days and thereafter gradually decreased, indicating that *Fgf16* expression was induced after the induction of *Fgf2* expression in the heart.

### Fgf2 stimulates Fgf16 expression in cultured neonatal rat cardiomyocytes

We examined *Fgf16* and *Fgf2* expression in cultured neonatal rat cardiomyocytes and noncardiomyocytes. *Fgf16* was more abundantly expressed in cardiomyocytes than noncardiomyocytes (Fig. 4E). In contrast, Fgf2 was more abundantly expressed in noncardiomyocytes than cardiomyocytes (Fig. 4F). Low *Fgf16* and *Fgf2* expression levels in cultured noncardiomyocytes and cardiomyocytes might reflect the possibility of cross-contamination of one cell versus the other, respectively. We also examined the effect of Fgf2 on *Fgf16* expression in both cells. Fgf2 stimulated *Fgf16* expression in cultured cardiomyocytes but not noncardiomyocytes (Fig. 4E). In addition, we also examined the effect of Fgf16 on *Fgf2* expression in both cells. However, Fgf16 did not affect *Fgf2* expression in both cells (Fig. 4F).

## Discussion

Fgf16 acts as a local paracrine signaling molecule. *Fgf16* expression levels are much more abundant at adult stages than at embryonic stages, indicating potential roles of Fgf16 in the heart at adult stages (Hotta *et al*. 2008; Lu *et al*. 2008a). However, as heart function examined by echocardiography is essentially normal in *Fgf16* knockout mice at adult stages (Hotta *et al*. 2008), the roles of Fgf16 in the adult heart remain unclear.

The renin–angiotensin system is a key mediator of cardiac adaptations to hemodynamic overload. In hypertension, the heart responds to increased afterload by initiating adaptive remodeling processes, including cardiac hypertrophy and fibrosis (Rosenkranz 2004). Although these structural alterations represent the heart's efforts to maintain systolic function, they are deleterious over time and ultimately result in progressive heart failure. Angiotensin II induces cardiac hypertrophy and fibrosis (Rosenkranz 2004). To examine pathophysiological roles of Fgf16 in the adult heart, we examined the heart of *Fgf16* knockout mice injected with angiotensin II.

### Fgf16 contributes to a myocardial environment that protects against hypertrophy and fibrosis

Systolic blood pressure tends to be increased in *Fgf16* knockout mice with angiotensin II infusion. In addition, dilated cardiomyopathy is also slightly induced in *Fgf16* knockout mice with angiotensin II infusion. Compensatory cardiac failure response to angiotensin II is promoted in *Fgf16* knockout mice. Histological analysis indicates that angiotensin II-induced cardiac hypertrophy and fibrosis are significantly promoted in *Fgf16* knockout mice. Increased expression levels of marker genes for cardiac hypertrophy and/or fibrosis also support promoted angiotensin II-induced cardiac hypertrophy and fibrosis in *Fgf16* knockout mice. These observations suggest that endogenous Fgf16 contributes to a myocardial environment that protects against hypertrophy and fibrosis, at least when the stress is induced by Angiotensin II.

### Tgf-β_1_ may promote angiotensin II-induced cardiac remodeling in *Fgf16* knockout mice

Tgf-β_1_ critically involved in the pathogenesis of cardiac hypertrophy and fibrosis. Angiotensin II induces cardiac hypertrophy and fibrosis by up-regulation of *Tgf-β*_*1*_ expression via the angiotensin II type 1 receptor in cardiac myocytes and fibroblasts. Induction of *Tgf-β*_*1*_ is absolutely required for angiotensin II-induced cardiac hypertrophy and fibrosis in mice, indicating that Tgf-β_1_ acts downstream of angiotensin II (Rosenkranz 2004). Cardiac *Tgf-β*_*1*_ expression levels in *Fgf16* knockout mice with angiotensin II infusion are significantly higher than those in wild-type mice. These observations are consistent with a requirement for Tgf-β1 signaling in the promotion of angiotensin II-induced cardiac hypertrophy and fibrosis in *Fgf16* knockout mice.

### Different responses to cardiac hypertrophy and fibrosis in *Fgf16* and *Fgf2* knockout mice

Although most *Fgf* genes have been disrupted by gene targeting in mice, cardiac phenotypes at adult stages have been shown in only *Fgf2* knockout mice (Itoh & Ornitz 2011). Cardiac hypertrophy and fibrosis were less developed in *Fgf2* knockout mice with myocardial infarcts (Virag *et al*. 2007). Furthermore, isoproterenol-induced cardiac hypertrophy was protected in *Fgf2* knockout mice (House *et al*. 2010). The cardiac phenotypes of *Fgf2* knockout mice are apparently opposite to that of *Fgf16* knockout mice reported here.

### Possible mechanism of Fgf16 action in cardiac hypertrophy and fibrosis

*Fgf16* is expressed mainly in cardiomyocytes. Fgf16 is efficiently secreted and acts as a paracrine signaling molecule (Miyake *et al*. 1998; Itoh & Ornitz 2011). In contrast, Fgf2 is mainly expressed in noncardiomyocytes. The biochemical properties of Fgf2 are also distinct from those of Fgf16. Fgf2, which has not a secretory signal sequence, is not a typical secretory protein. Fgf2 might be released from damaged cells or by an exocytotic mechanism that is independent of the endoplasmic reticulum–Golgi pathway (Nickel 2010). Fgf2, which is stored in these cells, is released in response to a hemodynamic stress (Clark *et al*. 1995; Kaye *et al*. 1996).

The phenotype of *Fgf16* knockout mice indicates that Fgf16 probably prevents angiotensin II-induced cardiac hypertrophy and fibrosis by repressing *Tgf-β*_*1*_ expression in mice. The role of Fgf16 is apparently opposite to that of Fgf2, which promotes them, indicating that the role of Fgf16 in cardiac remodeling is clearly distinct from that of Fgf2. Although Fgf16 does not induce *Tgf-β*_*1*_ expression in cultured cardiomyocytes and noncardiomyocytes, Fgf16 antagonizes Fgf2-induced *Tgf-β*_*1*_ expression in both cells. *Fgf16* expression is induced after the induction of *Fgf2* expression in the heart. In cultured cardiomyocytes, *Fgf16* expression is induced by Fgf2. In contrast, *Fgf2* expression is not affected by Fgf16 in cultured cardiomyocytes and noncardiomyocytes. There are seven major Fgfr proteins (Fgfrs 1b, 1c, 2b, 2c, 3b, 3c and 4) with differing ligand-binding specificity (Beenken & Mohammadi 2009; Itoh & Ornitz 2011). Among these *Fgfr*s, the heart predominantly expresses *Fgfr1c* (Fon Tacer *et al*. 2010). Fgf16 competes with Fgf2 for the binding site for Fgfr1c (Lu *et al*. 2008b). These results suggest a possible mechanism whereby Fgf16 probably prevents angiotensin II-induced cardiac hypertrophy and fibrosis by competing with Fgf2 for the binding site for Fgfr1c.

## Experimental procedures

### Animal experiments

Wild-type and *Fgf16* knockout mice on a C57BL/6 background were maintained in a light-controlled room and allowed free access to a normal diet (Hotta *et al*. 2008). Only male mice were used for experiments. Our ethics committee specifically approved this study. All animal studies were conducted in accordance with principles by the Animal Research Committee of Kyoto University Pharmaceutical Sciences, based on International Guiding Principles for Biomedical Research Involving Animals.

### Angiotensin II infusion

Mice at 10 weeks of age were subcutaneously implanted with an osmotic minipump (Alzet model 2002, Alza Corp) to continuously infuse angiotensin II in 10 mM acetic acid at a dose of 1.44 μg/g per day or an identical volume of 10 mM acetic acid as vehicle.

### Echocardiography

Mice at 12 weeks of age infused for 14 days were examined by conscious echocardiography. During the echocardiography, the animals were restrained by grasping the skin on the back of the neck and wrapping the tail (Xu *et al*. 2007). Heart rate, LVIDd, LVIDs, FS, EF, IVSTd and LVIDs were calculated using an echocardiographic system (Toshiba Power Vision 8000) equipped with a 12-MHz imaging transducer (Nakanishi *et al*. 2007). Systolic blood pressure was measured in conscious mice at 12 weeks of age using a noninvasive computerized tail-cuff method (Kuwahara *et al*. 2010).

### Histological analysis

The heart was fixed overnight in 10% formaldehyde, dehydrated, embedded in paraffin and sectioned at 6 μm. Sections stained with Masson's trichrome were examined by light microscopy. Images of the heart sections were captured. Cardiomyocyte sizes were quantitatively determined with Image J software. Blue-stained interstitial fibrotic areas were also quantitatively determined with Image J software.

### Expression analysis by RT-qPCR

cDNA was synthesized from RNA extracted from the heart. The cDNA was amplified by qPCR (Hotta *et al*. 2008), using the following primers: mouse/rat *18S rRNA* (sense primer, 5′-CCA ACG TCT GCC CTA TCA ACT T-3′; antisense primer, 5′-CCG GAA TCG AAC C CT GAT T-3′), mouse *Anp* (sense primer, 5′-TTC TTC CTC GTC TTG GCC TTT-3′; antisense primer, 5′-GAC CTC ATC TTC TAC CGG CAT CT-3′), mouse *Bnp* (sense primer, 5′-CAC CGC TGG GAG GTC ACT-3′; antisense primer, 5′-GTG AGG CCT TGG TCC TTC AAG GTC ACT-3′), mouse *βMhc* (sense primer, 5′-ATG TGC CGG ACC TTG GAA-3′; antisense primer, 5′-CCT CGG GTT AGC TGA GAG ATC A-3′), mouse *Col1a* (sense primer, 5′-CGA AGG CAA CAG TCG CTT CA-3′; antisense primer, 5′-GGT CTT GGT GGT TTT GTA TTC GA-3′), mouse *Ctgf* (sense primer, 5′-AGC AGC TGG GAG AAC TGT GT-3′; antisense primer, 5′-GCT GCT TTG GAA GGA CTC AC-3′), mouse *Postn* (sense primer, 5′-AAC CAA GGA CCT GAA ACA CG-3′; antisense primer, 5′-TGT GTC AGG ACA CGG TCA AT-3′), mouse *Mmp2* (sense primer, 5′-TTT GCT CGG GCC TTA AAA GTA T-3′; antisense primer, 5′-CCA TCA AAT GGG TAT CCA TCT C-3′), mouse *Tgf-β*_*1*_ (sense primer, 5′-CTG CGC TTG CAG AGA TTA AA-3′; antisense primer, 5′-GAA AGC CCT GTA TTC CGT CT-3′), rat *Tgf-β*_*1*_ (sense primer, 5′-CTG CGC CTG CAG AGA TTC AA-3′; antisense primer, 5′-GAA AGC CCT GTA TTC CGT CT-3′), moue/rat *Fgf16* (sense primer, 5′-CTG ATC AGC ATC AGG GGA GT-3′; antisense primer, 5′-AGG TGG AGG CAT AGG TGT TG-3′), moue *Fgf2* (sense primer, 5′-AGC GAC CCA CAC GTC AAA CT-3′; antisense primer, 5′-CGT CCA TCT TCC TTC ATA GCA AG-3′) and rat *Fgf2* (sense primer, 5′-GAC GGC TGC TGG CTT CTA AGT-3′; antisense primer, 5′-TTT CCG TGA CCG GTA AGT GTT-3′). *18S* rRNA levels were used as an internal control.

### Cell culture

Cardiomyocytes and noncardiomyocytes were prepared from apical halves of cardiac ventricles from Wistar rats at 1 or 2 days of age (Nakagawa *et al*. 1995) and plated at a density of 3.5 × 10^4^ cells/cm^2^ in gelatin-coated 24-well culture dishes (Becton Dickinson) in Dulbecco's modified Eagle's medium (DMEM) supplemented with 10% fetal bovine serum, 100 U/ml penicillin G and 100 μg/ml streptomycin. After a 40-h incubation, the cells were maintained in serum-free DMEM for 10 h. After a preconditioning period, the cultures were incubated in serum-free DMEM containing 1 mg/ml BSA with 1 ng/ml recombinant Fgf2 and/or 25 or 100 ng/ml recombinant Fgf16 (Danilenko *et al*. 1999) for 40 h. cDNA was synthesized from RNA extracted from cultured cardiomyocytes. *Tgf-β*_*1*_, *Fgf2* and *Fgf16* expression levels were examined by qPCR as described above.

### Statistical analysis

Results are expressed as the mean ± standard error of measurement (SEM). The statistical significance of differences in mean values was assessed with Student's *t*-test.
